# Supplementary figures and images for: Condensin IDC, DPY-21, and CEC-4 maintain X chromosome repression in C. elegans
Source: PLoS Genet. 2025 Apr 9;21(4):e1011247. doi: 10.1371/journal.pgen.1011247 (PMC12013946; doi:10.1371/journal.pgen.1011247)

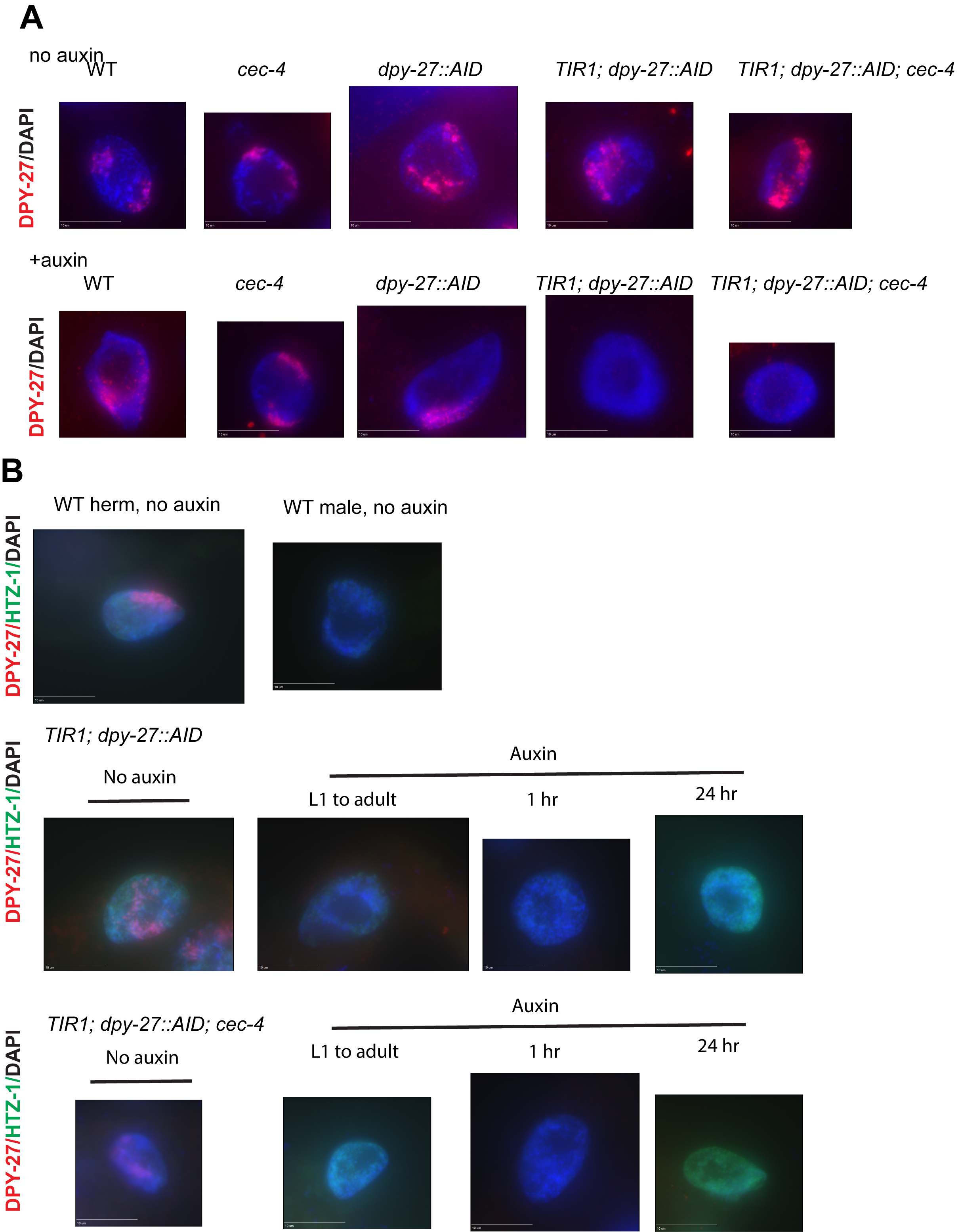

Supplement: S1 Fig — (A) Unmodified original images of panels shown on Fig 2A. DPY-27 IF is shown in red, DNA stain (DAPI) in blue. (B) Unmodified original images of panels shown on Fig 2B. DPY-27 IF is shown in red, HTZ-1 IF in green (used as staining control) and DNA stain (DAPI) in blue. The main figure only shows that DPY-27 and DAPI color channels. Scale bars, 10 µm. (TIF) [file pgen.1011247.s001.tif]

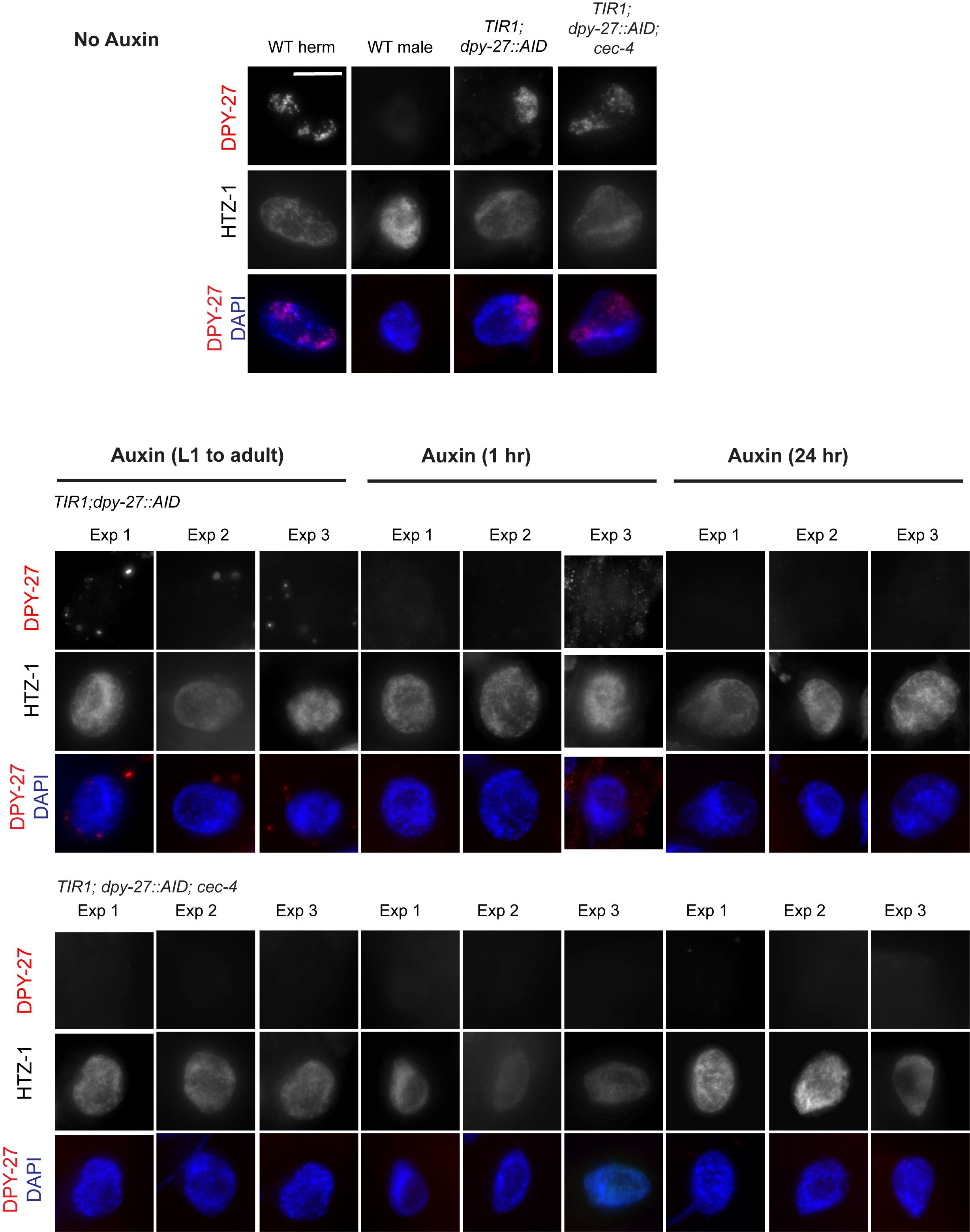

Supplement: S2 Fig — Control non-auxin treated worms and worms treated with auxin from the L1 stage from 3 days, or from day 1 of adulthood for 1 hr, or 24 hours were dissected and stain with anti-DPY-27 antibody, and anti-HTZ-1for staining control. Results from three independent experiments are shown. The merged images show DPY-27 in red and DNA (DAPI) in blue. Scale bars, 10 µm. (TIF) [file pgen.1011247.s002.tif]

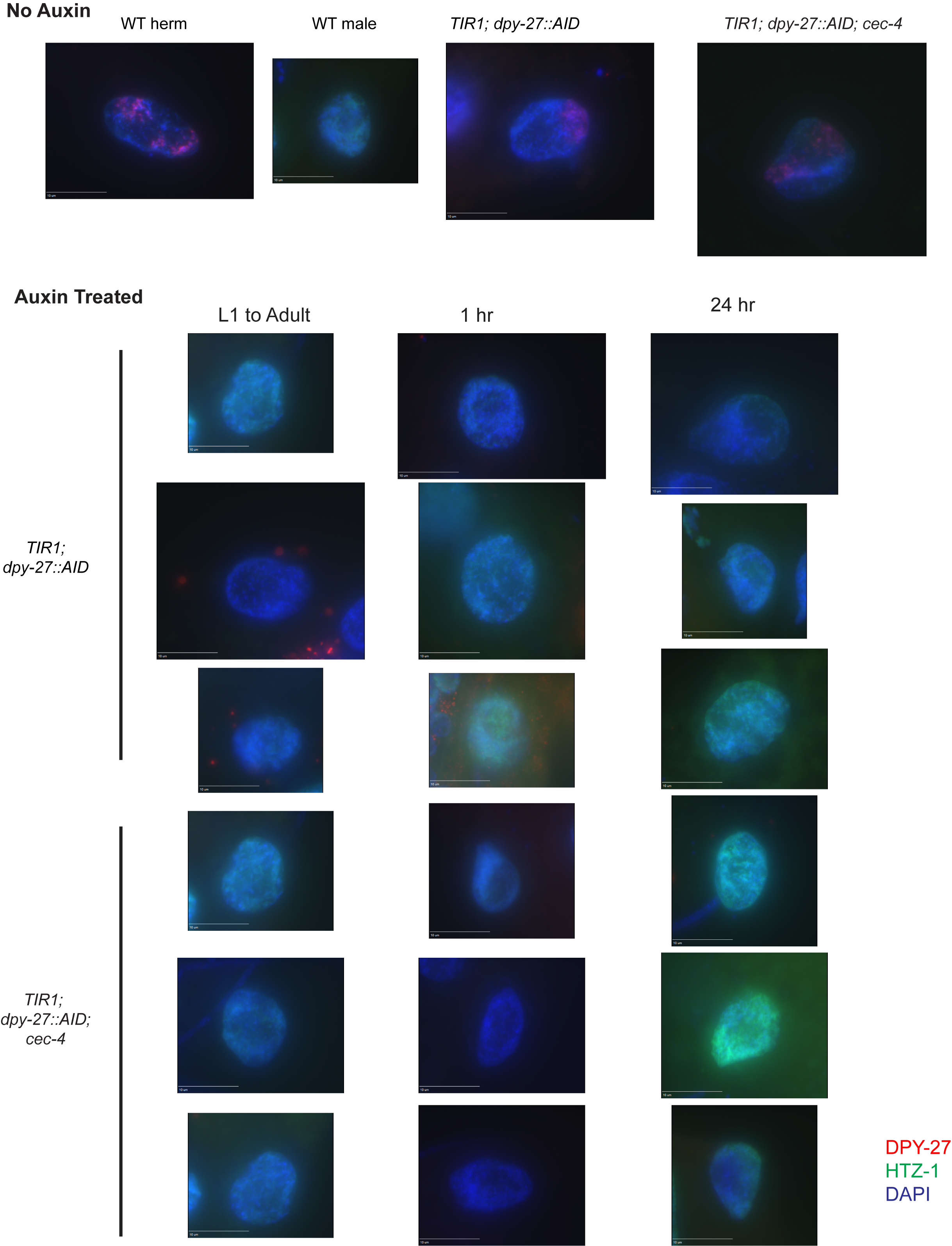

Supplement: S3 Fig — DPY-27 is shown in red, HTZ-1 (staining control) in green, and DNA (DAPI) in blue. Scale bars, 10 µm. (TIF) [file pgen.1011247.s003.tif]

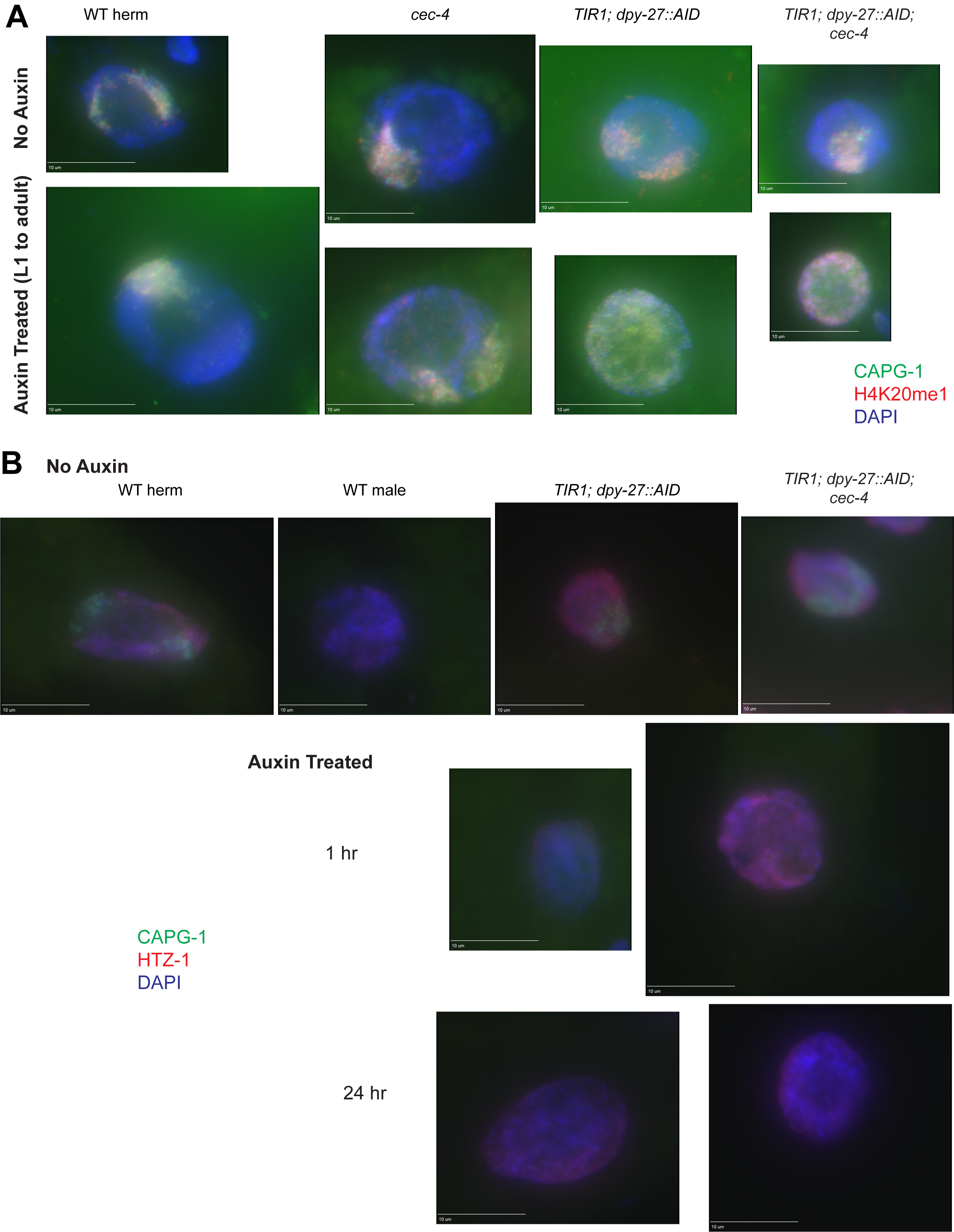

Supplement: S4 Fig — (A) Unmodified images for Fig 3A. CAPG-1 staining is shown in green, H4K20me1 staining in red, DNA (DAPI) in blue. The main figure only shows the CAPG-1 and DAPI channels. (B) Unmodified images for Fig 3B. CAPG-1 staining is shown in green, HTZ-1 (staining control) in red, and DNA (DAPI) in blue. The main figure only shows the CAPG-1 and DAPI channels. Scale bars, 10 µm. (TIF) [file pgen.1011247.s004.tif]

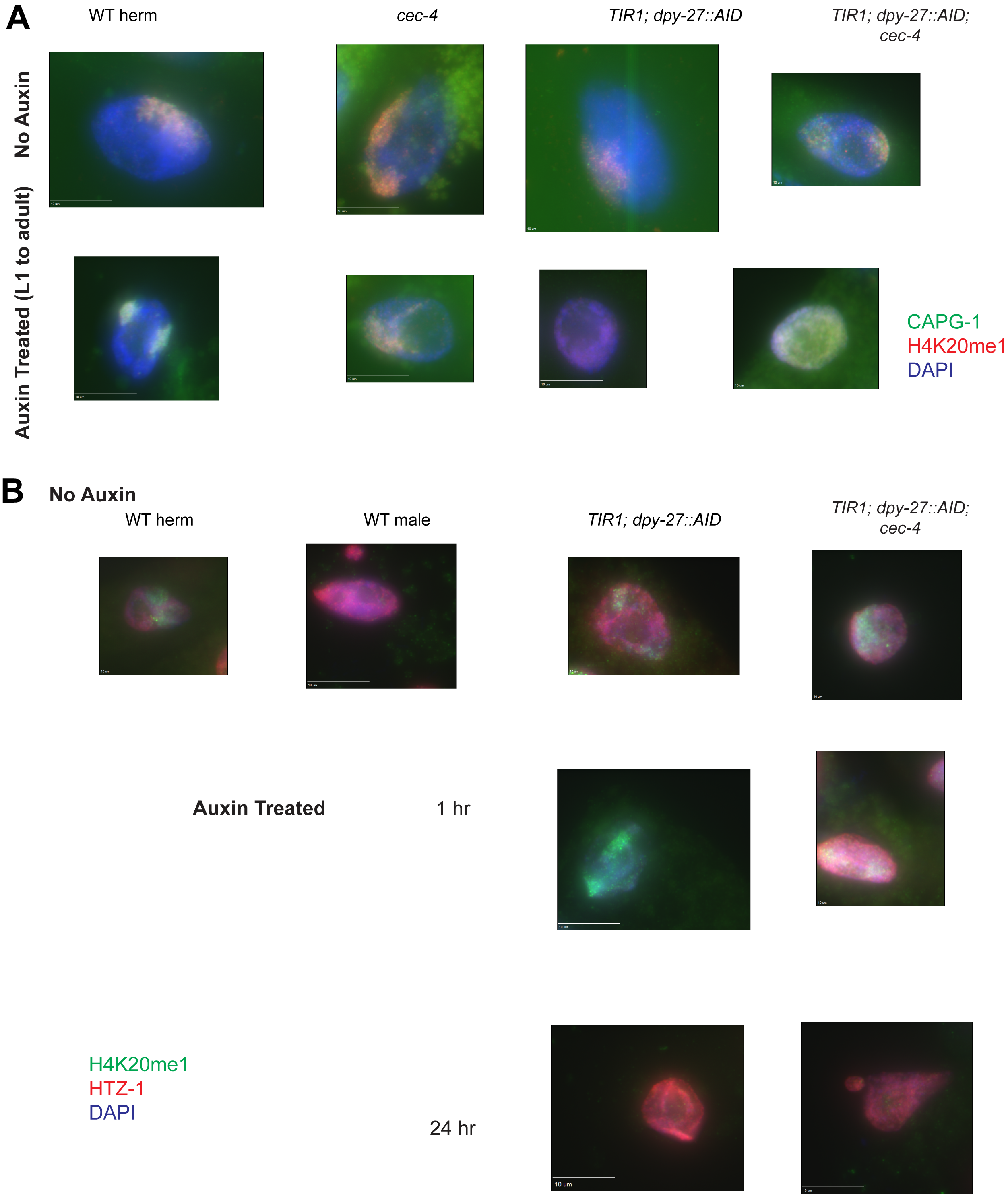

Supplement: S5 Fig — Fig 4. (A) Unmodified images for Fig 4A. CAPG-1 staining is shown in green, H4K20me1 staining in red, DNA (DAPI) in blue. The main figure only shows the H4K20me1 and DAPI channels. (B) Unmodified images for Fig 4B. H4K20me1 staining is shown in green, HTZ-1 (staining control) in red, and DNA (DAPI) in blue. The main figure only shows the H4K20me1 and DAPI channels. Scale bars, 10 µm. (TIF) [file pgen.1011247.s005.tif]

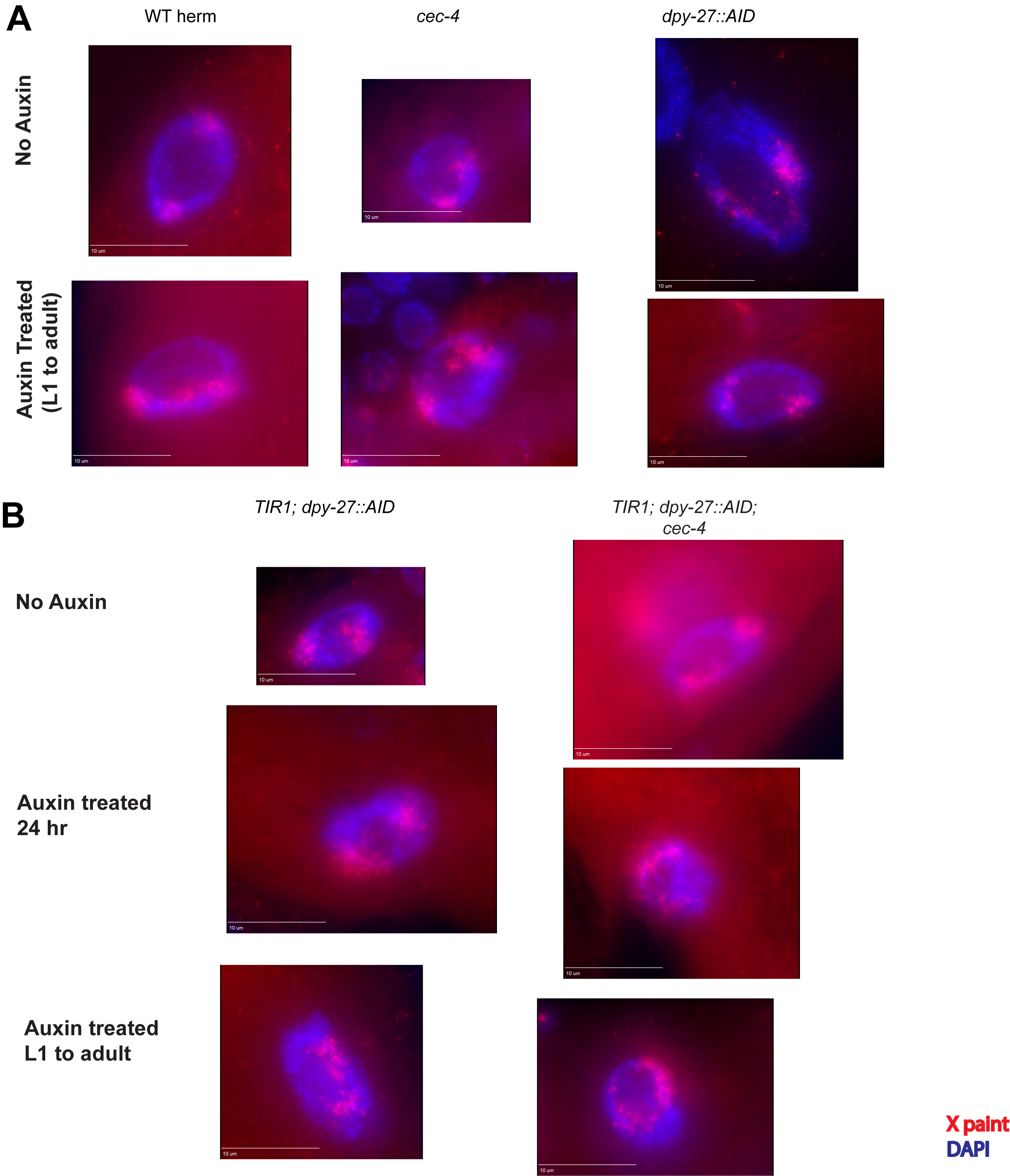

Supplement: S6 Fig — Fig 5. X chromosome paint FISH probe is shown in red and DNA (DAPI) in blue. Scale bars, 10 µm. (TIF) [file pgen.1011247.s006.tif]

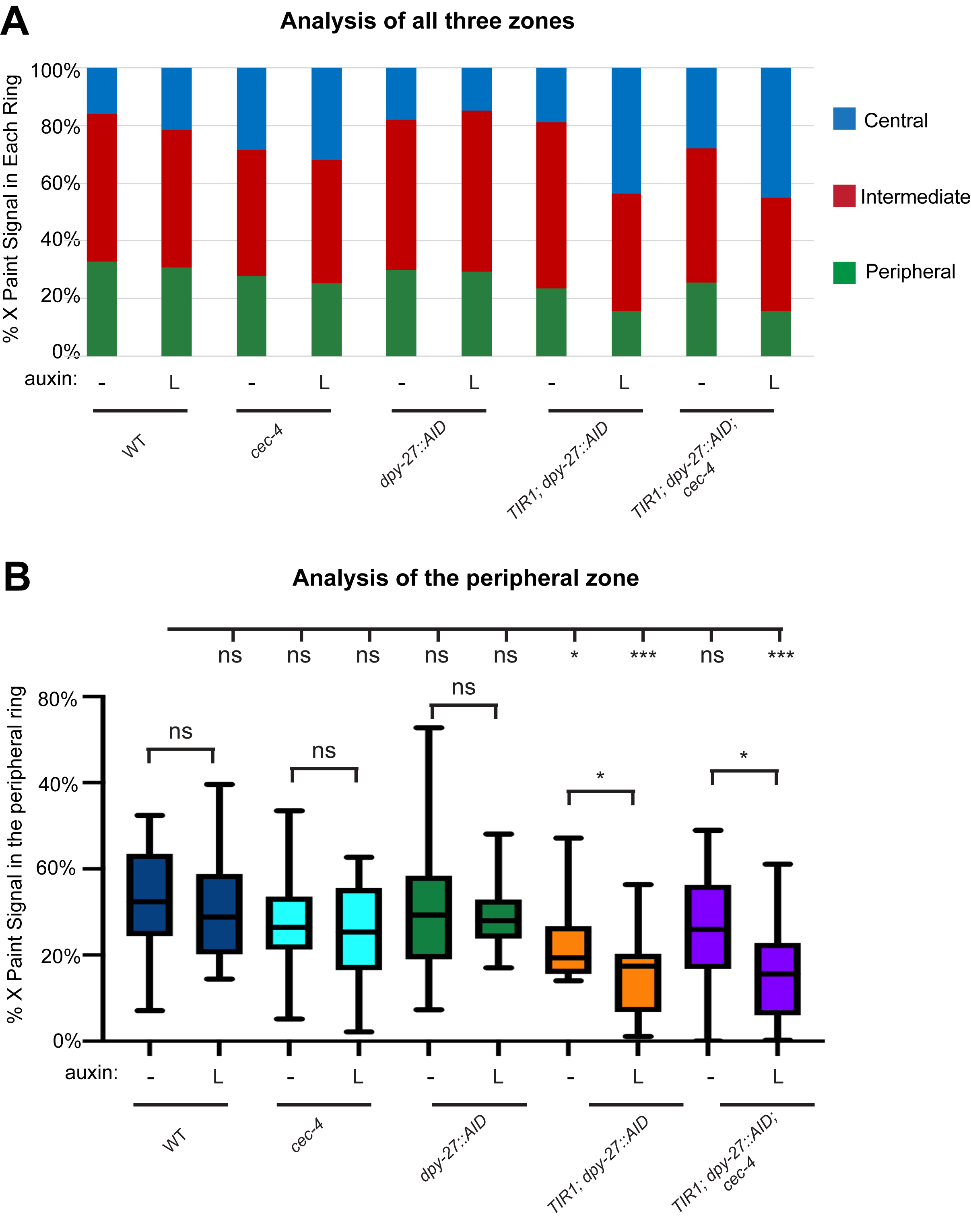

Supplement: S7 Fig — (A) Three-zone assay for the X paint FISH signal in strains grown without auxin (-) or after auxin treatment from L1 larval stage to adulthood (L) showing all three concentric zones. Fig 5 shows only the data for the innermost zone. (B) The proportion of the X paint signal seen in the peripheral zone of the nucleus. Differences between samples were evaluated using unpaired Student’s t-test. For complete statistical analysis see S7 File. n.s. = not significant, * = p < 0.05, ** = p<0.01, *** = p < 0.001. (TIF) [file pgen.1011247.s007.tif]

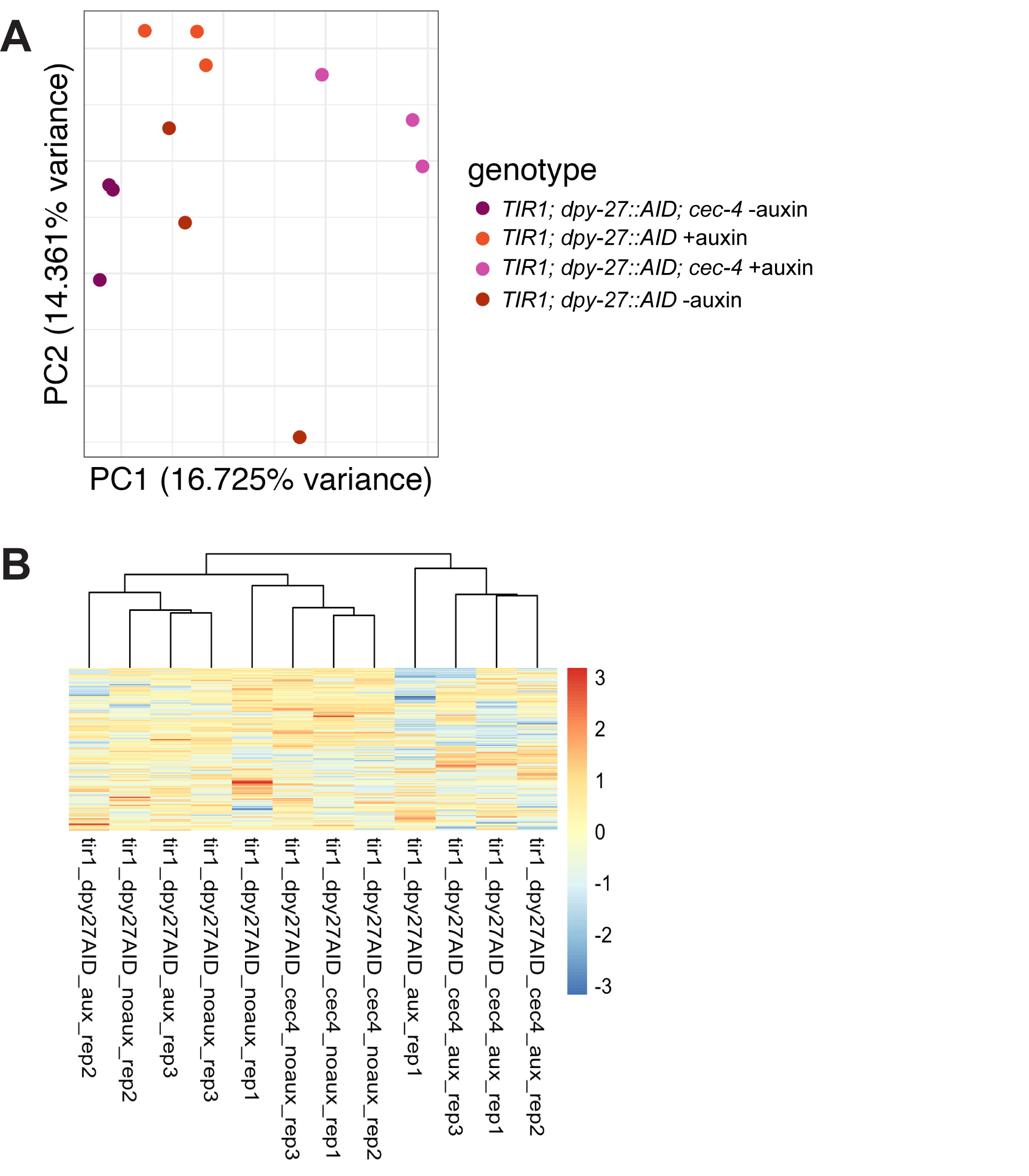

Supplement: S8 Fig — (A) Principal component analysis (PCA) plot depicting the relationships among TIR1; dpy-27::AID, and TIR1; dpy-27::AID; cec-4 with and without auxin treatment. (B) Unsupervised hierarchical clustering of TIR1; dpy-27::AID, and TIR1; dpy-27::AID; cec-4 with and without auxin treatment using Manhattan distance to calculate distance between samples, and ward.D2 algorithm to cluster samples. (TIF) [file pgen.1011247.s008.tif]

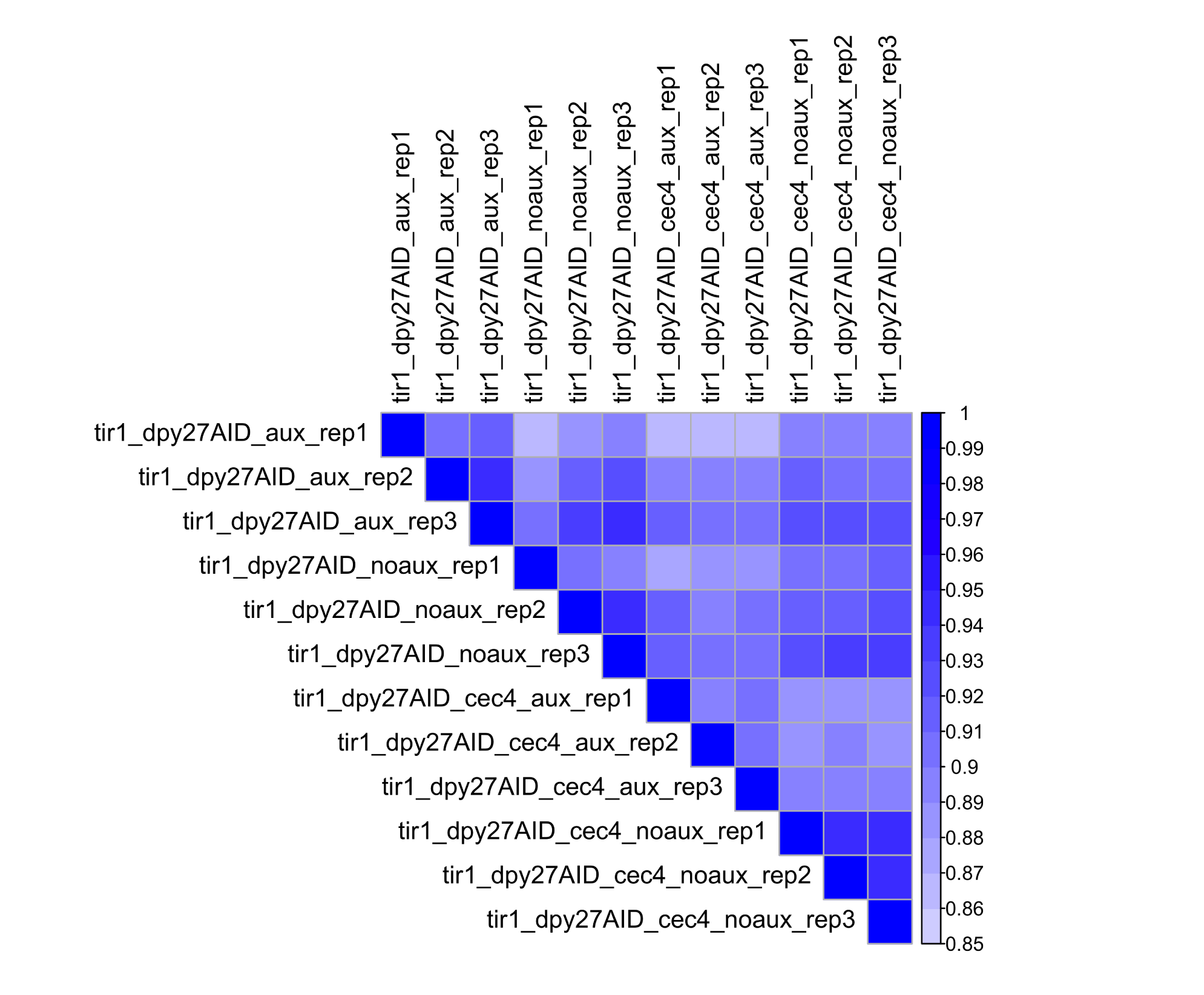

Supplement: S9 Fig — Heatmap represents color corresponding to the Pearson correlation coefficient between any two samples. Samples analyzed included the TIR1; dpy-27::AID, and TIR1; dpy-27::AID; cec-4 strains with and without auxin treatment. (TIF) [file pgen.1011247.s009.tif]

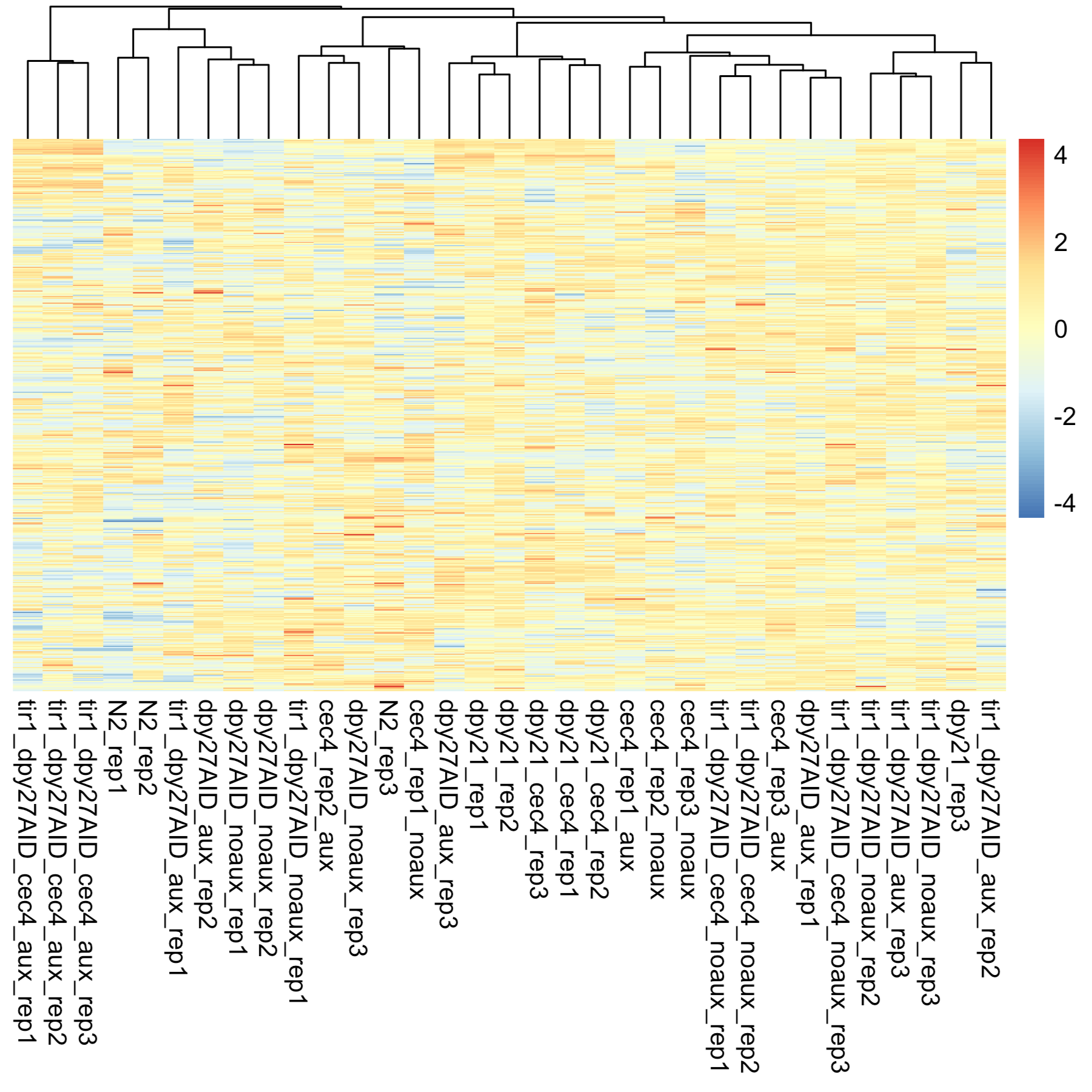

Supplement: S10 Fig — Unsupervised hierarchical clustering of all RNA-seq using Manhattan distance to calculate distance between samples, and ward.D2 algorithm to cluster samples. (TIF) [file pgen.1011247.s010.tif]

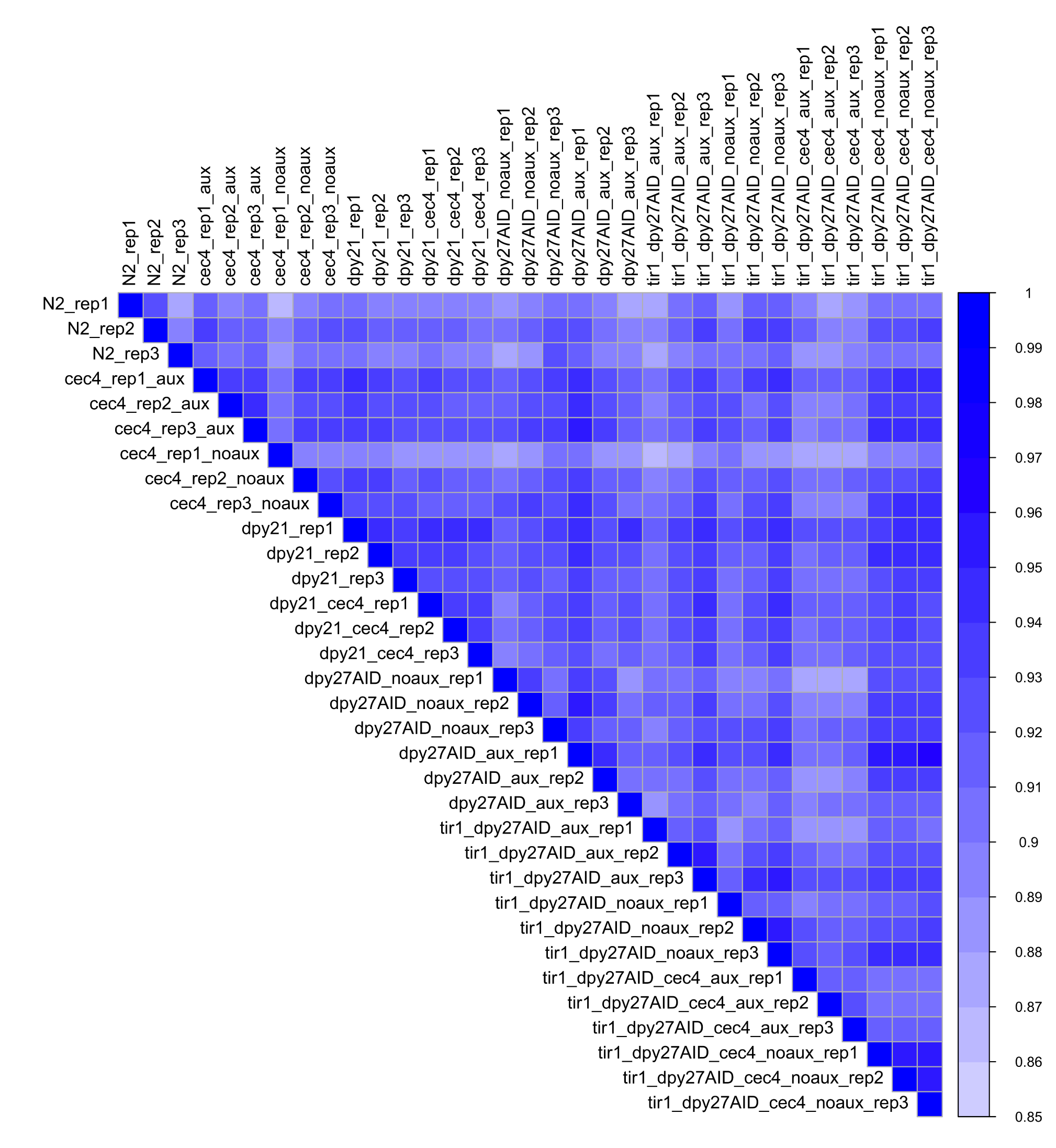

Supplement: S11 Fig — Heatmap represents color corresponding to the Pearson correlation coefficient between any two samples. (TIF) [file pgen.1011247.s011.tif]

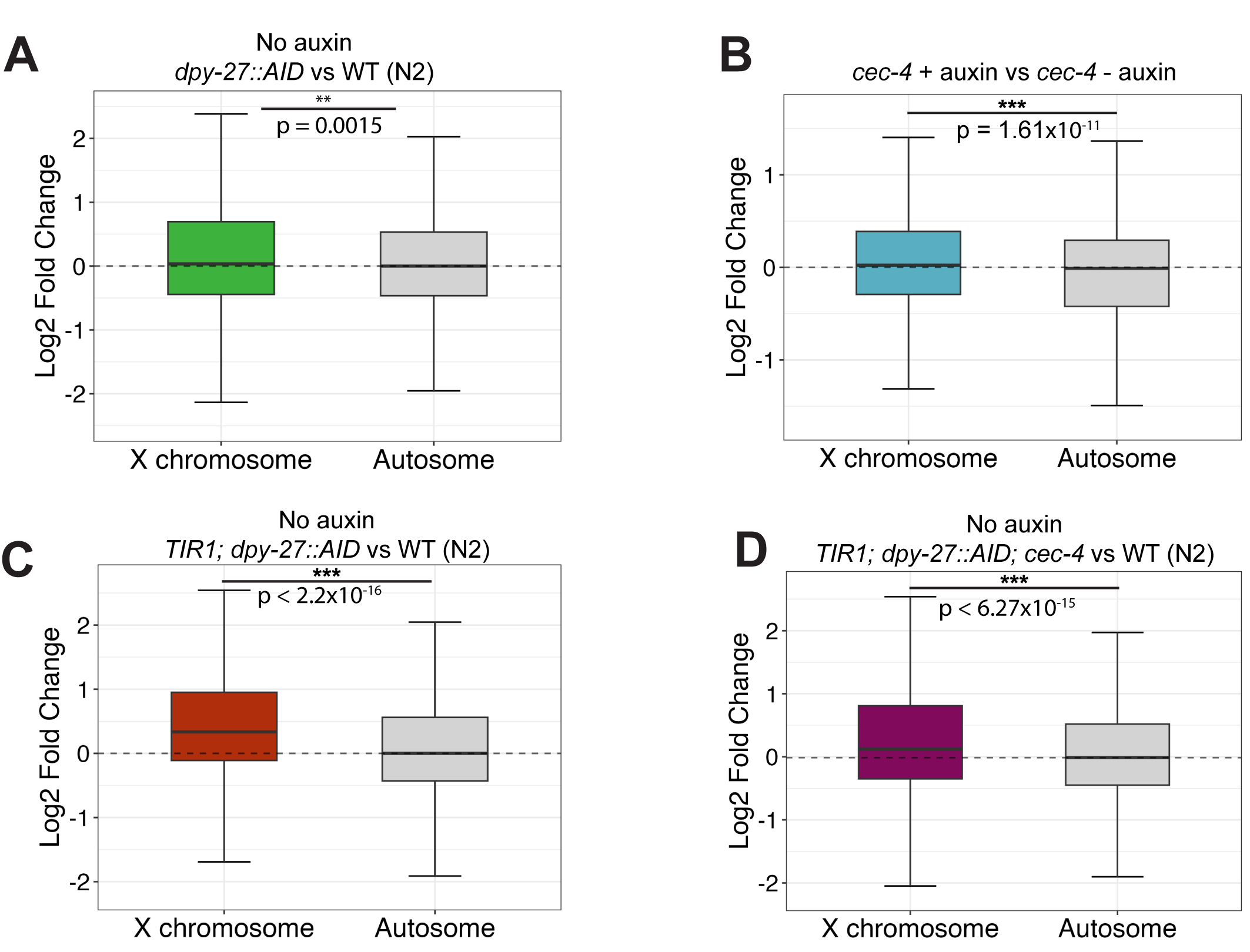

Supplement: S12 Fig — (A-D) Boxplots depicting the distribution of the expression difference of X-linked genes and expression difference of genes on autosomes. Gene expression is plotted as the log2 ratio of the strains being compared. Statistical significance is determined by the differences in gene expression between the X and autosomes by a Wilcoxon rank-sum test. Samples compared are shown above the boxplots (n.s. = not significant, * = p < 0.05, ** = p<0.01, *** = p < 0.001). (TIF) [file pgen.1011247.s012.tif]

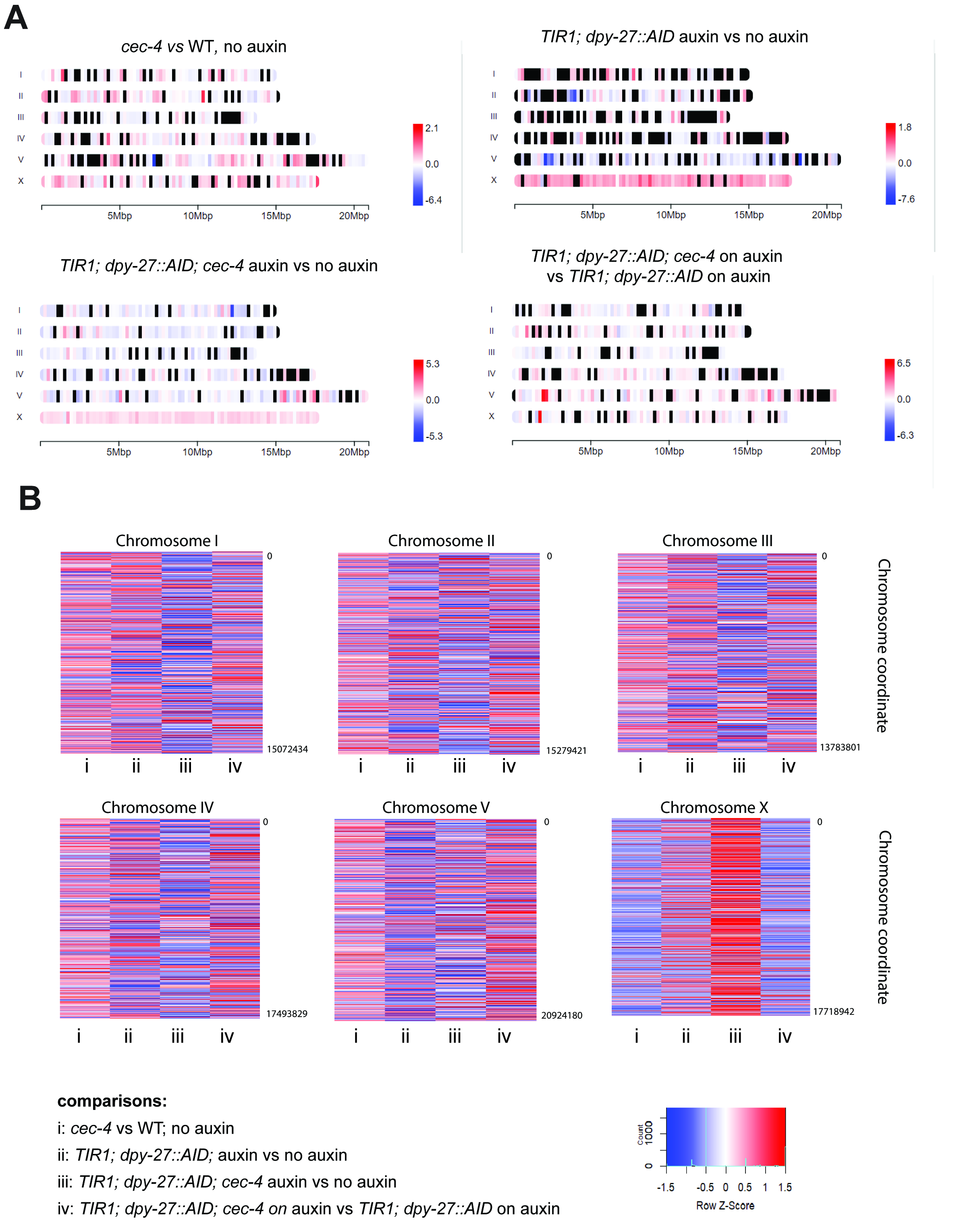

Supplement: S13 Fig — (A) Chromomap analysis of log2 fold change in gene expression along the X chromosomes and autosomes. Samples compared are indicated above the maps. (B) Heatmap of log2 fold changes along each chromosome arranged by chromosomal coordinates. Comparisons are indicated at the bottom. (TIF) [file pgen.1011247.s013.tif]

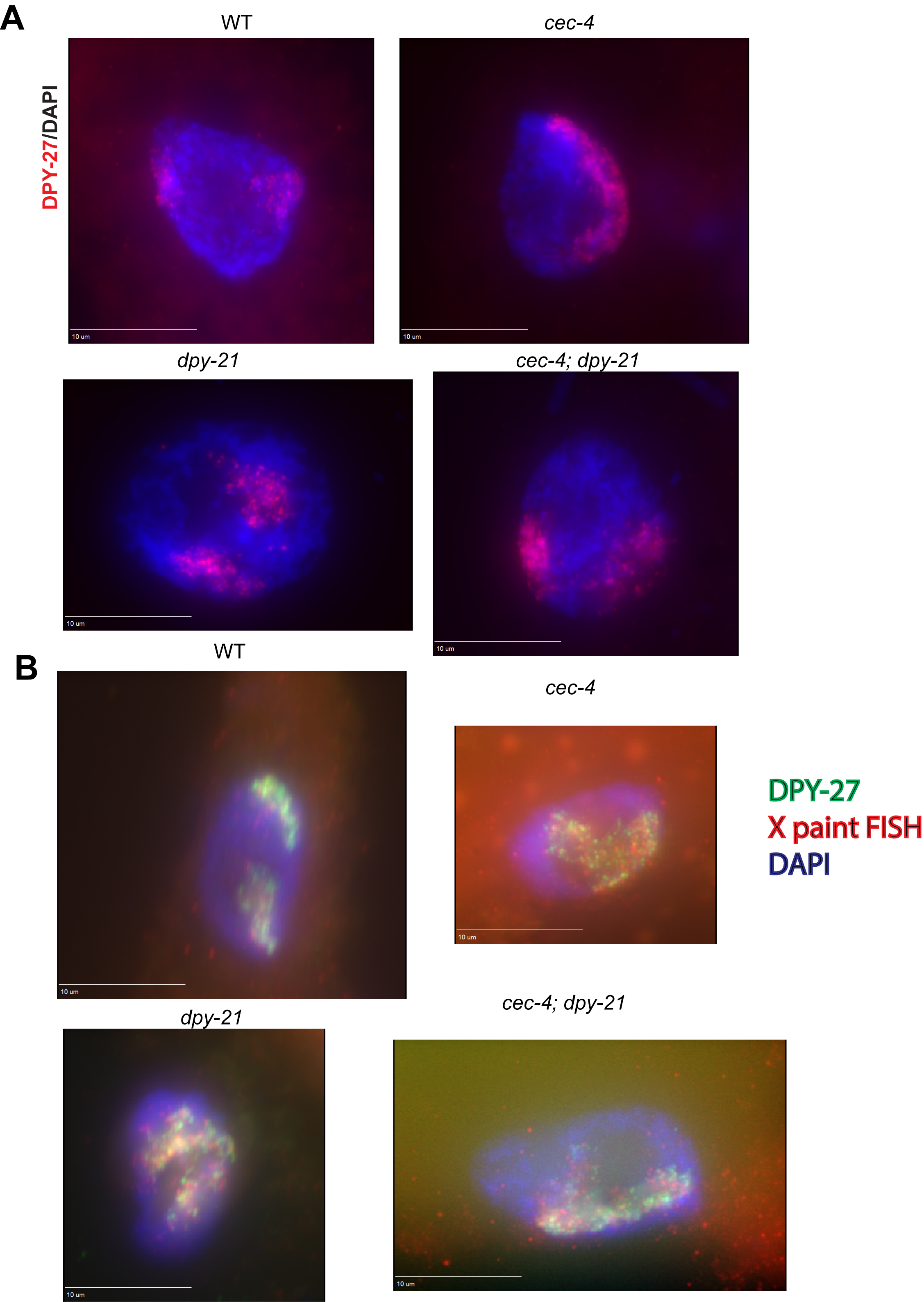

Supplement: S14 Fig — (A) Original unmodified images for Fig 7E. DPY-27 IF staining is shown in red and DNA (DAPI) in blue. (B) Original unmodified images for S15 Fig. DPY-27 IF is shown in green, X chromosome paint FISH probe is shown in red and DNA (DAPI) in blue. Scale bars, 10 µm. (TIF) [file pgen.1011247.s014.tif]

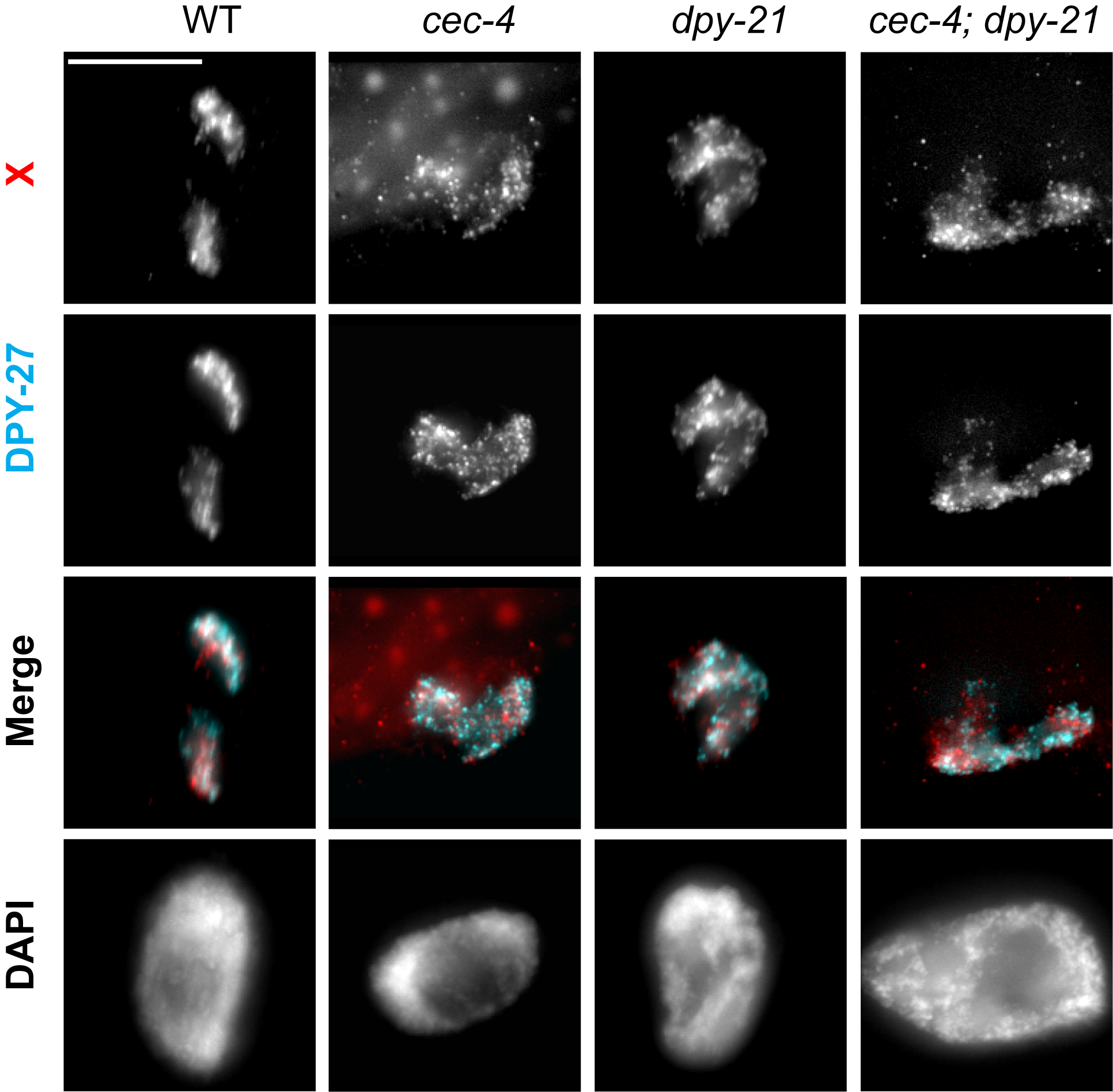

Supplement: S15 Fig — . Signal for DPY-27 IF colocalizes with X-paint FISH signal. In the merged image, DPY-27 is shown in blue and X paint FISH signal in red. DAPI is shown in grayscale. Scale bars, 10 µm. (TIF) [file pgen.1011247.s015.tif]

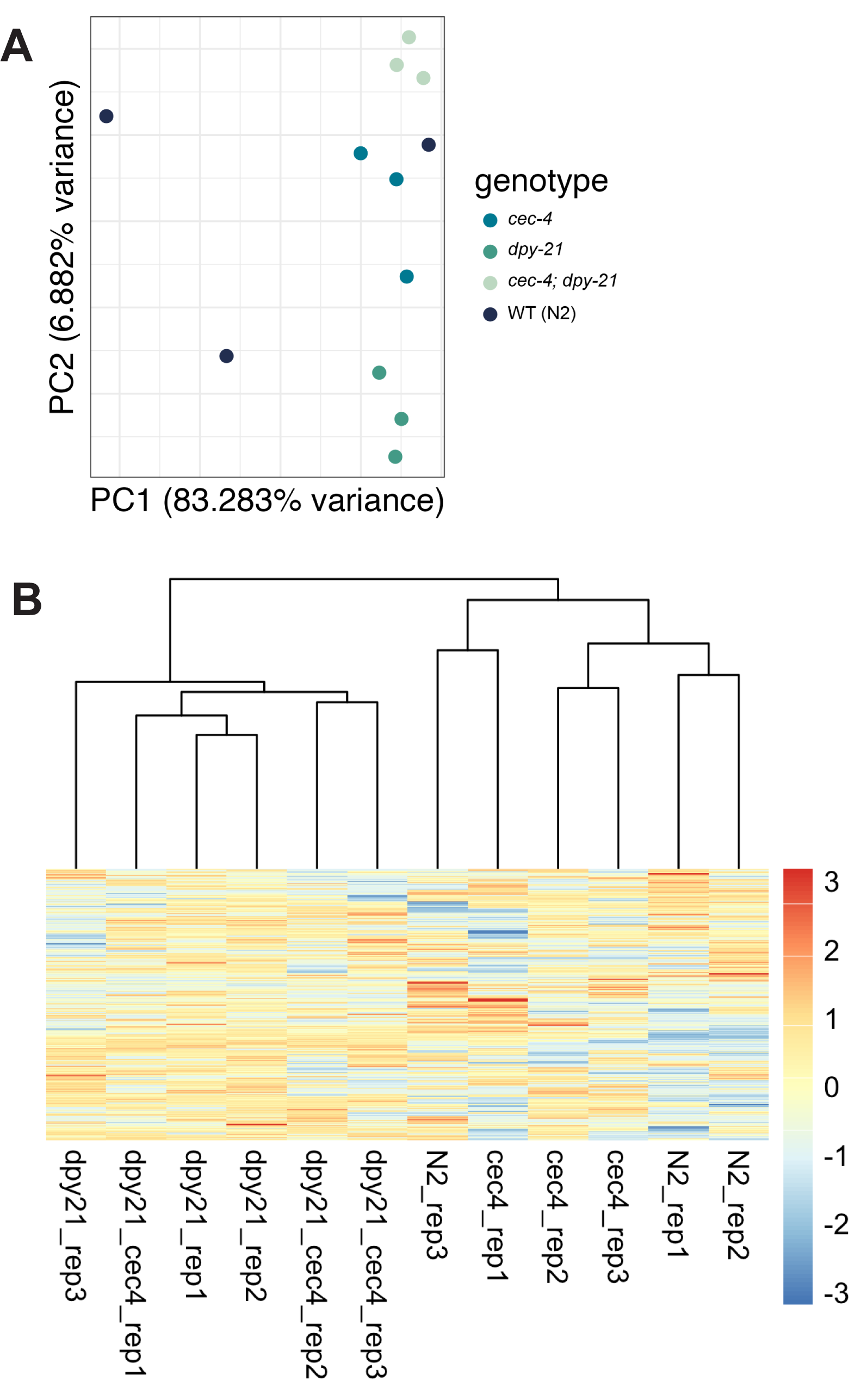

Supplement: S16 Fig — (A) Principal component analysis (PCA) plot depicting the relationships among wild-type, single mutants (cec-4 and dpy-21), and double mutants (cec-4; dpy-21) based on gene expression profiles. (B) Unsupervised hierarchical clustering of cec-4 and dpy-21 mutant RNA-seq datasets using Manhattan distance to calculate distance between samples, and ward.D2 algorithm to cluster samples. (TIF) [file pgen.1011247.s016.tif]

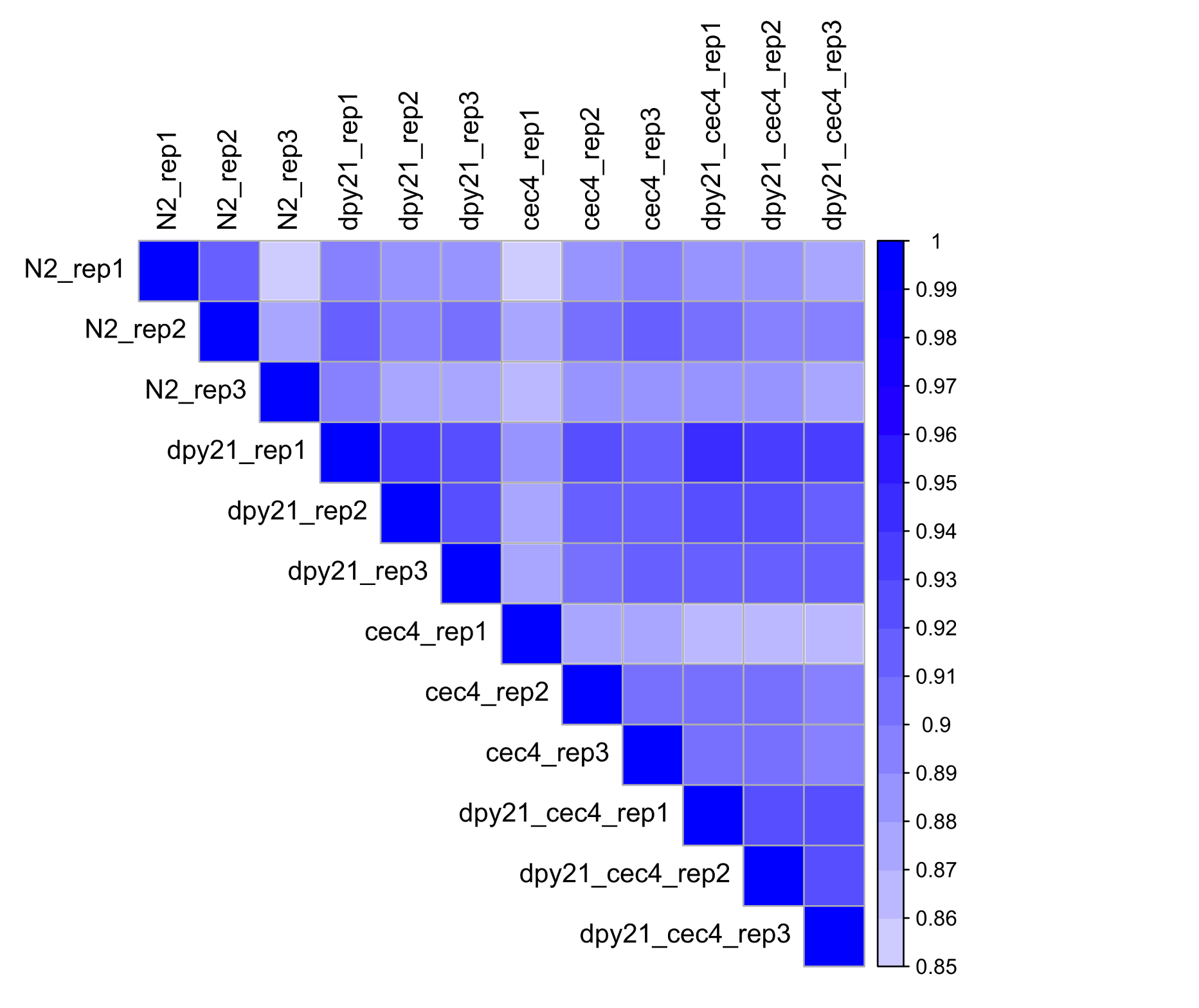

Supplement: S17 Fig — . Heatmap represents color corresponding to the Pearson correlation coefficient between any two samples. (TIF) [file pgen.1011247.s017.tif]
