# Supplementary material for: Condensin IDC, DPY-21, and CEC-4 maintain X chromosome repression in C. elegans
Source: PLoS Genet. 2025 Apr 9;21(4):e1011247. doi: 10.1371/journal.pgen.1011247 (PMC12013946; doi:10.1371/journal.pgen.1011247)
Supplement: Attachment — Submitted filename: Response_Trombley.docx [file pgen.1011247.s029.docx]

Dear Editors and Reviewers,

Thank you for the feedback on our manuscript. We believe we addressed most of the concerns raised by the reviewers and we hope that you will find the new version much improved. The additional experiments we performed and changes we made to the figures and text are detailed below. In summary, the revised version has three figures with new data as well as new analysis on most figures and 14 additional supplementary figures.

The editors specifically pointed out issues with the low sample size and biological replicates, as well as the lack of quantification of images, as major concerns. We were also asked to provide correlation and clustering plots to evaluate quality of RNAseq data. As detailed below in the response to specific reviewer comments, these changes were made. Below you will find the reviewers comments in black and our responses in blue.

We generated a private reviewer token for GSE262451. It is: iladugumbdwdpar

We also provide a text file which changes from the previous version of the manuscript tracked. The file is a bit messy, as a significant amount of new text has been added, a large portion of the original text was revised, and other sections were moved around.

Reviewer #1: Trombley et al., characterize the role of condensing IDC at different stages of development in C. elegans. They show that while this complex, required for dosage compensation, is essential during embryogenesis, it is dispensable for survival in post-embryonic stages. To determine this, they make use of the auxin inducible degradation system, which allows to rapidly deplete a protein of interest, in their case DPY-27, upon exposure to the plant hormone auxin. They also investigate the role of a non-condensin DCC subunit termed DPY-21, which is a histone demethylase responsible for generating high levels of H4K20me1 on the repressed X chromosome in hermaphrodites, and of CEC-4, a H3K9me perinuclear anchor. The biological question addressed in this work is interesting and the genetic tools developed by the authors look convincing and suitable to their aims. However, the study at the current stage is very preliminary and requires substantial experimental work in order to become solid and convincing. Below, we outline the major points that in our opinion need to be addressed.

1. The approach of the authors relies mostly on immunostainings and X chromosome paints. Yet, all of the presented microscopy results come from a single biological replica where only about 20 nuclei per condition were analyzed. This is absolutely not sufficient to draw any meaningful conclusion. The authors should repeat their experiments to reach at least 3 biological replicas with the number of nuclei analyzed that should reach at least 50 in total. A possibility could be to quantify 15-20 nuclei per biological replica.

We apologize for not making it clear in our original submission the number of replicates performed. In the original submission, we already had data from at least three independent experiments. In each experiment we dissect about 20-30 individual worms. During the dissection process, part of the gut spills out and some portion of it separates sufficiently from the rest of the tissue to image individual nuclei. Some worms fall off the slides, in others the gut is not sufficiently separated from the rest of the tissue, and we are usually able to image no more than 3 worms per slide. We never image more than 3 nuclei per worm, and often image no more than 1. Therefore, the original data came from 3+ independent experiments, and between 7-15 individual worms. Power analysis indicates that for most of the differences we observe, a sample size of 16-20 is sufficient. In the amount of time we had available for the revision (including the extension granted by the editors), we had to prioritize what to focus on. Our main focus was to generate the new data and new quantifications the reviewers requested. We also boosted the samples size in some of our experiments where the original sample size was on the lower end. We note that with the number of nuclei analyzed in the current submission, some of our data points are statistically significantly different, even when we believe no biological difference exists (for example, new Figure 3C).

For the revision, we performed three independent experiments for the quantifications shown on new Figures 2-4. We also performed additional replicates of the FISH experiments to boost the n values for genotypes where the numbers were lower (new Figure 5). We were unable to reach a sample size of 50, especially for the FISH experiments, where due to the harsher treatment involved in FISH, even collecting images of 20 nuclei of sufficient quality (i.e. suitable for quantification) quantified required 3-4 independent experiments. Again, even with this samples size the differences we report are statistically significant.

2. Besides the major issue raised in point 1, some of the microscopy data are not quantified at all. For example:

- In Fig. 2 A, B, C. no quantification of the degradation of DPY-27 is shown.

To quantify depletion of DPY-27, we performed three independent experiments, and we imaged 38-50 individual nuclei total. To be able to quantify signal across samples, we standardized exposure times. (Since our original images were not collected with uniform exposure times, we had to re-do these experiments and collect new data.) We included WT males in the analysis, as they do not express DPY-27, and they were used as controls for background staining by the antibody. These experiments show that within 1 hr of auxin treatment, DPY-27 levels decrease to what is seen in males. These data are shown on new Figure 2.

Likewise, there is no quantification of CAPG-1 and H4K20me1. The authors write in the text that the signals of these factors become diffused. However, it is impossible to distinguish between a diffused signal vs loss of signal in the images presented. A quantification of the total fluorescent intensity/ per nucleus is needed to claim diffusion over degradation. Furthermore, it is possible that the signal decreases AND is diffused.

In our original submission, we did not mean to distinguish between diffuse signal and no signal. We simply wanted to state that these proteins/modifications are no longer enriched on the X chromosomes. However, for the revision, we did perform new experiments the reviewers suggested and we can now make this distinction. We used with standard exposure times, based on what was used in WT, to allow this type of quantification. We again performed three independent experiments. We quantified the images two different ways. First, we measured enrichment of the signal on the X as compared to autosomes. Second, we quantified total fluorescence signal. The quantification shows that although CAPG-1 signal intensity remains somewhat higher than the signal seen in males, its enrichment on the X is no longer detectable after 1 hr. After 24 hours of auxin treatment, the signal intensity also becomes similar to that of males. The results indicate that the CAPG-1 signal first becomes diffuse and then it decreases, suggesting that the protein does degrade, but not as fast as DPY-27. However, it does not appear to localize to the X as soon as DPY-27 is degraded. For H4K20me1, the changes are slower. After 1 hr, enrichment is still detectable in nuclei, but not after 24 hr. Total signal intensity is less clear, since we observed a large cell-cell variation in signal intensity, and most genotypes and treatments are not different from WT hermaphrodites. These data are shown on new Figures 3 and 4.

- In Fig. 4 A,B. Again there is no quantification of DPY-27 degradation. Also, there is no quantification of CAPG-1 and H4K20me1. The authors should do a colocalization analyses to prove that the effect on the distribution of these factors is partial after 1h on auxin.

The figures were reorganized. These data are now included in new Figures 2, 3, and 4.

- Fig.6 F, the authors state that loss of CEC-4 or DPY-21 “does not influence the localization of DPY-27 to the X”. However, there is no quantification for this statement. From the image presented, it looks like DPY-27 is more diffused in dpy-21 mutants. Thus, to draw meaningful conclusions, a colocalization analysis should be conducted.

Previous studies by us (Snyder et al PLoS Genetics 2016) and others (Brejc et al Cell 2017) showed that neither *cec-4* nor *dpy-21* impact DCC localization. Thus, this is not a new conclusion we wish to make. The study by Brejc et al and an additional study (Anderson Dev Cell 2019) used DPY-27 staining to measure the volume of the X chromosome in these mutants. Thus, these experiments were essentially a control and we moved them to a supplemental figure. We also replaced the images with more representative images.

The reason why we prefer to use DPY-27 IF staining instead of X paint FISH is that IF has better signal to noise ratio and requires less harsh treatment compared to FISH, and thus we can obtain more accurate measurements. Obviously, for the strains in which we induce degradation of DPY-27, this technique cannot be used, and therefore we have to resort to FISH.

3. In Fig.3, the authors perform a chromatin positioning assay quantifying the amount of X chromosome found in three concentric nuclear zones of equal surface. Such an assay does not directly address detachment of the X (more precisely the left tip of the X chr, see Ikegami et al., 2010) from the nuclear lamina as the current assay can be greatly influenced by X chromosome compaction, which is indeed affected in some of their conditions. For example, it is possible that the X chromosome, visualized with a whole chromosome paint, remains bound to the nuclear lamina but due to decompaction, reaches at least partially the internal zone of the nucleus. To be able to draw conclusions about the subnuclear distribution, or perinuclear positioning, of the X chromosome, the authors should use a different approach, such as LEM-2ChIP qPCR or LMN-1 or EMR-1 DamID amplifying sequences on the X, or, to stick to microscopy, a DNA FISH with a probe specifically targeting only the left tip of the X-chromosome, followed by the 3 zones assay or a measure of the distance from the nuclear envelope.

We previously performed the assays suggested by the reviewer using probes specific to left, middle, and right ends of the chromosome (Snyder et al PLoS Genetics 2016). The results were unexpected and showed that the left end (the one which is most enriched for LEM-2 ChIP signal) remains near the nuclear lamina in cec-4 and dpy-27 mutants, and instead the middle portion of the X is most affected. The fact that the left remains near the nuclear lamina is consistent with results from others which showed that CEC-4 is required to anchor heterochromatic regions to the nuclear lamina in embryos, but not in differentiated tissues of larvae (Gonzalez-Sandoval et al Cell 2015). In more differentiated tissues, it is believed that in differentiated tissues additional anchors keep these regions near the nuclear lamina.

Why the middle of the X is more impacted in *cec-4* mutants is somewhat of a mystery, and we agree with the reviewer that some of this relocalization could be due to the decondensation of the chromosome, particularly decondensation of the gene-rich middle domain (Snyder et al PLoS Genetics 2016). We included discussion of these data in the revised manuscript.

4. For all of the RNAseq data in Fig5 and 7, the authors should provide, as supplementary material, analyses showing the correlation and clustering between biological replicas.

PCA plots were included in the original submission. Now we also added clustering and correlation analyses in supplementary figures S9-S11 and S16 and S17.


5. For the analyses of Figs. 2, 3, 5 and 7 the authors compare worms that are likely at different developmental stages or worms with a different cell and nuclear size (as they show in Fig. 1D, that depletion of DPY-27 results in a stunted growth). While this might still be ok to allow drawing conclusions about the expression of X-linked genes vs autosomal genes (Figs 5 and 7), the ploidy and/or nuclear volume of animals exposed to auxin or not should be determined for the microscopy analyses. If the ploidy/nuclear volume ratio is the same between auxin and no auxin, then the samples can be compared. In contrast, different ploidy/nuclear volume ratios do not allow to conclude what determines the change in the amount of volume occupied by the X chromosome.
On this line, a proper positioning assay, as suggested in point 3, would instead not be affected by differences in ploidy/nuclear volume ratios as the authors would have a direct read out of the position of a smaller locus.

In all of our analysis we normalize the volume of the X chromosome to nuclear volume. We do not report volume in cubic microns, but rather as the percentage of the nuclear volume occupied by the X chromosomes. The same way differences in ploidy would not affect the positioning assay because it compares the location of the chromosome to a portion of the nucleus, ploidy/nuclear volume differences should also not affect the percentage of the nuclear volume occupied by the X chromosome.

In addition, we see similar levels of decondensation when auxin treatment is started in adulthood (see Figure 5), after the worms were allowed to fully develop and presumably reach WT ploidy levels and the analyzed cells have become fully differentiated and postmitotic. These data argue that are conclusions are not affected by potential differences in ploidy, if any indeed exists.

6. The role of the H3K9me reader CEC-4 in the dosage compensation process remains unclear. For example, in Fig. 4B, the authors show that H4K20me1 is still present on the X chromosome, yet the X chromosome is decompacted, as they previously showed, and in Fig. 3. A quantitative, ChIPseq (or qPCR targeting X-linked vs autosomal genes) approach is required to determine whether, despite being still present in IF, the levels of H4K20me1 are reduced or not in absence of CEC-4. To gain further mechanistic insights, the authors should also test the levels of H4K20me2/3 and H3K9me2/3 in presence and absence of cec-4 and dpy-21, with H3K9me2/3 being directly bound by CEC-4.

The main goal of the current study was to investigate the role of DPY-27 and the requirement for its continued presence on the X chromosome after dosage compensation has been established. We feel that the experiments suggested by the reviewer are beyond the main scope of this study. All available evidence indicates that CEC-4 functions with H3K9me2/me3 (papers from the Gasser lab) and at least at the level of microscopy, CEC-4 has no impact on H4K20me1. Conversely, DPY-21 is responsible for the X enrichment of H4K20me1 (Brejc et all, Cell 2017), but there is no evidence that it has any impact on H3K9me2/3. Rather, these two pathways appear to be separate and independent. In fact, our data on Fig 4 supports that hypothesis. A mutation in *cec-4* does not influence how fast enrichment of H4K20me1 is lost in the absence of DPY-27. While we agree that a potential crosstalk between the H4K20me1/DPY-21 and H3K9me/CEC-4 pathways have not been addressed rigorously, we believe such analysis belongs in a separate study.

Minor points:
1. No auxin and auxin treatment should be in the same panel, as they need to be compared.

These changes were made on all figures.

2. We find the choice of not using asterisks but A, B, C etc to indicate statistically significant differences rather confusing.

Using letters to indicate statistical differences can quickly convey the message about all pairwise comparisons. We remade our figures with asterisks, but it made the figures rather busy despite not showing all comparisons we were able to convey with letters. For full statistical analysis and all comparisons, the reader now has to go to Supplemental files.

3. We suggest that the authors consider using boxplots rather than bar plots to better represent the variability of the data, particularly the microscopy ones.

All graphs quantifying microscopy data now use boxplots with the whiskers indicating min-max.

Reviewer #2: In this study, Trombley et al. investigate the genetic requirements of X-chromosome dosage compensation during the maintenance phase in C. elegans animals. The authors focus on the roles of dosage compensation complex component DPY-27 and the chromodomain protein CEC-4 in intestinal cells. They find that loss of DPY-27 and CEC-4 in the maintenance phase lead to X-chromosome decompaction, H4K20me1 loss, and gene expression de-repression, but animals are still viable. The authors conclude that the DCC and lamina tethering consolidate X-chromosome repression in larvae. These findings are of interest to the chromatin biology community in general and dosage compensation field specifically.
To strengthen the conclusions of the manuscript and allow readers to assess the robustness/reproducibility of the data, I suggest the following:

- The experiments of Fig. 1 should have side-by-side quantification of the efficacy of DPY-27 depletion. DPY-27 stains should be performed on the same batches of embryos/larvae that phenotypes are quantified. How variable is the auxin depletion in the different conditions tested? Images from multiple replicates should be presented in the supplemental.

Auxin mediated depletion is very robust and reproducible. On new supplemental figure 2, we show data from three independent replicates and in all cases DPY-27 levels were indistinguishable from background within one hour of initiating auxin treatment. Depletion is quantified on main Figure 2, based on worms collected in three independent experiments.

As for the counts presented on Figure 1, we cannot go back now and perform IF on the same batch of worms that were used for the three replicates of viability counts. In addition, imaging of dead embryos and larvae would result in low quality images. However, we routinely did the opposite for all remaining experiments: we used impacts on viability/phenotype as a readout to make sure that the depletion works (i.e. our auxin plates are not too old).

- All figure legends should state the number of biological and technical replicates performed.

This information is added to all figure legends. The information is also available is supplemental files of numerical data and statistical analysis.

- For all imaging data, uncropped and unaltered raw images should be provided in the supplemental along with their metadata. Some images in the figures appear to be over-processed, and the microscopy section of the methods is under-described. Exposure times and laser/LED power should be reported. All processing steps should be included – especially choice of nuclei to display, and all images should be displayed with identical min-max intensities per assay to have accurate comparisons. These values should be reported.

We consulted with the editors about how to go about providing uncropped/unaltered images. All of our images are 3D image stacks and providing the unaltered original data would require providing all Z planes. The editors suggested that we provide maximum intensity projections, rather than all focal planes and that we do not need to provide all metadata. These unaltered original images are now provided as Supplemental Figures for all main figures with microscopy images. (We note that these unaltered images are RGB, because that is how the original images were acquired). We only provide one representative image (the same one as on the main figure) and we do not provide all images used for quantification (up to 50 nuclei per condition). We ended up remaking all of our figures, simply because it was easier to pick one of the 20-50 images rather than trying to identify the original image used in the original submission. We also provided more details on image processing in the methods section. For images in which we quantified fluorescence intensity, we used the same exposure times and performed identical contrast enhancements (i.e. identical min-max intensities per assay).

Colorblind-friendly lookup tables should be used (e.g. do not combine red and green).

All of our images now use either red/blue or red/cyan colors (except for the original unaltered images which remain RGB). We also provide grayscale images of the color channel being quantified.

- For the gene expression analysis, it would be informative to dig a little deeper and un-collapse the gene expression plots to show how expression changes over the length of the X and autosomes. Do the regions overlap with H4K20me1? Known DCC ChIP regions?

We mapped gene expression changes onto coordinates of chromosomes (new Supplemental Figure 13). This analysis did not show any preference for derepression on the chromosomal domain attached to the nuclear lamina (i.e. the left end). This is consistent with our prior analysis of *cec-4* and H3K9me mutants, where we also did not see preferential gene depression in this domain (Snyder et al PLoS Genetics 2016).

Previous studies also analyzed correlations between DCC peaks, H4K20me1 peaks and gene expression changes. But no such correlation was found. DCC binding does not correlate with more significant changes in gene expression in mutants (Jans et al Genes and Dev 2009, Kruesi et al eLife 2013). Similarly, changes in TAD structure in dosage compensation mutants does not correlate with gene expression change (Crane et al Nature 2015). Instead DCC and H4K20me1 peaks appear to correlate with transcription levels (rather than magnitude of impact on gene expression change in mutants (Ercan et al Curr Biol 2009, Vielle et al PLOS Genetics 2012, Kramer et al PLoS Genetics 2015). The impact of these mechanisms are thought to be more chromosome-wide, rather local influences near ChIP peaks. Discussion of this topic was added to the manuscript.

More details in the Methods section are required for ‘downstream analysis’ – which R packages? All scripts should be provided in supplemental/GitHub.

We included some more details, and scripts are also provide in GitHub.

- I am curious about the difference between intestinal cells and other cells of the animal – are the conclusions held true? The extremely high ploidy of intestinal cells could be a factor in the degree of X-chromosome decompaction and H4K20me loss. Can the authors compare to muscle/vulva/neuron nuclei in their images?

Unfortunately, to obtain the number of images we need for quantification, we are limited to intestinal cells. Our staining protocol relies on dissection, during which tissues spill out from the cuticle and thus became accessible for antibodies and FISH probes. The only tissues that “spill out” after dissection are the germline (which is not subjected to X chromosome dosage compensation) and the intestine. We occasionally see nuclei near the cut site also being stained, but not in sufficient numbers to do the kind of quantification we did for this study. In previous studies we were able to perform some limited analysis on non-intestinal cells (for example Lau et al Epigenetics and Chromatin 2014), and the conclusions appeared to hold true for other cells as well.

- Clarify the discussion regarding the role of CEC-4 in embryos vs larvae, discussing previous literature on cec-4 loss (i.e. Gasser lab work, etc.). It is still unclear to me what the model for CEC-4 function is in different stages, and the difference between autosomes and X chromosome role. Perhaps add a final model figure.

As specified in a response to reviewer 1, point 6, this discussion has been added to the paper. Since the role of CEC-4 remains unclear, we decided not to add a final model figure.
