# Supplementary material for: Condensin IDC, DPY-21, and CEC-4 maintain X chromosome repression in C. elegans
Source: PLoS Genet. 2025 Apr 9;21(4):e1011247. doi: 10.1371/journal.pgen.1011247 (PMC12013946; doi:10.1371/journal.pgen.1011247)
Supplement: Attachment — Submitted filename: Response.docx [file pgen.1011247.s030.docx]

Dear Editors,

Below please find our responses (in blue) to editor’s and reviewer’s comments (in black). We describe in detail all changes made in the text and figures. We look forward to hearing from you.

Editor requested changes:

Specifically, the use of the term "perinuclear tethering" seems inappropriate in that the data do not provide direct evidence of tethering per se as opposed to localization or positioning to the periphery as a consequence of compaction. Please change text in the title and body of the manuscript so as to not indicate direct evidence for tethering, although you may suggest it in a hypothetical model.

We changed the title and the text throughout to clarify that we can only draw conclusions about CEC-4 activity contributing to repression, not necessarily its role in perinuclear tethering. We only suggest in the discussion that the phenotypes may be related to CEC-4’s role in perinuclear tethering. The same logic applies to DPY-21 also. DPY-21 is known to be required for H4K20me1 enrichment, but it has additional uncharacterized functions. Technically, we cannot conclude that the impacts we see are due to lack of H4K20me1, but rather lack of DPY-21 activity. Therefore, in the title we changed “H4K20me” to “DPY-21” and “perinuclear tethering” to “CEC-4”.

**Reviewers’ comments to the Authors:
Please note here if the review is uploaded as an attachment.**

Reviewer #1: I appreciate the efforts the authors have made to refine the manuscript, making it more precise and convincing. The revised boxplots provide much clearer insights into the image quantifications, and overall, the manuscript has improved. The conclusions that dosage compensation is not essential for life during post-embryonic stages and that CEC-4 exacerbates the phenotypes of DCC-impaired animals are well-supported.

However, I still have significant concerns regarding the conclusion that DPY-27 is required for subnuclear localization (title of subparagraph in lines390-391 and Figure 5).

The section header has been changed to remove “and subnuclear localization”. Additional changes related to Fig 5 and Fig S7 are detailed below.

On page 12, lines 452, and Figure 5C, I appreciate the authors discussing the role of decondensation. Nonetheless, I remain unconvinced that the FISH-based monitoring of the whole X chromosome provides sufficient evidence to claim subnuclear relocation over decompaction. In Figures 5A-B, the authors clearly show that the compaction of the X chromosome is affected by the loss of both CEC-4 and DPY-27. In Figure 5C, they quantify the percentage of the X chromosome found in three zones of equal surface area. Changes in these distributions do not necessarily reflect positioning alterations; rather, the increase in centrally located X chromosomes are likely a result of decompaction, which allows the chromosome to occupy more internal regions.

In this paragraph, we changed the language to only state that is X is found in a more central location and that it could be due simply to dencondensation. We removed all active verbs such as claims about the DCC or CEC-4 positioning the X.

I remain convinced that, to substantiate their claims about perinuclear tethering (a term explicitly used in the title), the authors need to provide stronger evidence for their findings. As I have previously noted, they should focus on the left tip of the X chromosome, which is known to be anchored. While I understand the authors' response to my earlier comment that CEC-4 predominantly affects the middle part of the X and not the left tip (Snyder, 2016), this effect on the middle-X chromosome could still be explained by changes in compaction rather than positioning. What direct evidence is there, beyond the observation of a decompacted chromosome, that the X chromosome moves away from the nuclear periphery in any of their conditions? (less binding to lamin, LEM-2 or EMR-1?).The authors mention in one of their responses to my previous comments that CEC-4 does not affect positioning in larvae, as other mechanisms are at play in post-embryonic stages. This is true, but then I wonder: why are they testing CEC-4 and claiming a change in perinuclear anchoring in larvae and adults? Based on Cabianca et al 2019, CEC-4 has also a role in anchoring chromosomes in differentiated cells, which is mostly evident on endogenous chromatin with EMR-1-DamID. Thus, I don´t argue against their choice of testing it. But while the observed differences are indeed intriguing, these can most likely be attributed to decompaction rather than true changes in subnuclear positioning, especially in absence of any other complementary experimental test of binding to the nuclear envelope.

The title and the text have been changed accordingly to not make this claim.

Supplementary Figure 7 provides relevant data for Figure 5C, showing the distribution of the X chromosome across all three zones, which could help the authors make some conclusions on perinuclear positioning. First, to improve visualization, I suggest plotting the same genotypes with and without auxin side by side.

We changed the graph to show the same genotype with and without auxin treatment side by side.

For statistical analysis, I recommend conducting a chi-squared test to evaluate distribution changes across all zones, followed by a focused t-test to assess differences specifically in the outermost zone, similar to their approach for the central zone in the main figure. In fact, the critical question is: is there a statistically significant difference in the outer zone (perinuclear) when analyzed with a t-test? If a significant difference is observed, I might agree that perinuclear anchoring is involved, as if chromosome X leaves the outermost zone, even when decompacted, this may indeed indicate a change in localization, specifically a reduction in peripheral positioning. Without such evidence, the current data do not sufficiently support claims about perinuclear anchoring, subnuclear positioning, localization, or distribution and in that case, I would strongly recommend that the authors avoid describing their findings using these terms in their manuscript, unless additional evidence is provided. Even if the outermost zone occupancy is significantly different in DPY-27 perturbations, I suggest revisiting the title and removing the term "perinuclear tethering”, as some data would now be there, but not strong enough to appear in the title, as this could mislead the reader.

Our data is continuous, not categorical (expressed as a percent of the X signal in each zone). So a Chi-square test cannot be performed on our data. However, we did perform t-tests on the peripheral zone localization. DPY-27 depletion in fact did lead to statistically significant reduction in the signal in the peripheral zone, but a mutation in *cec-4* did not. This data is now shown in Fig S7B and discussed in the text. While this data does provide evidence that perinuclear tethering is involved (as pointed out by the reviewer), we still changed the text and the title and refer to CEC-4 activity, rather than perinuclear tethering.

I appreciate the effort made to provide additional analyses and quality control data for their RNA-seq. However, their statement in Lines 476-479 is unclear. Which ones are the more limited number of samples? Which genotype is not robustly changing? Did they anyway include them in the analyses or exclude them?

Our statement refers to the fact that there are very few gene expression changes in *cec-4* mutants, or in the *TIR1; dpy-27::AID* and *TIR1; dpy-27::AID; cec-4* strains without auxin treatment. These samples do not separate well from wild type (N2) in hierarchical clustering or Pearson correlation analyses. They were included in the analyses anyway, since the lack of gene expression change in these samples was expected. The more limited number of samples included the *TIR1; dpy-27::AID* and *TIR1; dpy-27::AID; cec-4* strains with and without auxin treatment (Figs S8 and S9). Separation between samples is clearer in this case. For example, PCA analysis shows that the auxin treated samples cluster away from non-auxin treated samples, and the auxin treated *TIR1; dpy-27::AID; cec-4* strain clusters away from the auxin treated *TIR1; dpy-27::AID* strain. This explanation is added to the text.

For the comparisons of *dpy-21, cec-4* and the double mutant, the analysis again shows that *cec-4* does not separate well from wild type, but the strains with *dpy-21* mutations do (Figs S16 and S17).

Suppl. Fig13: please make this figure also in a version where, across panels, the data are plotted with the same scale bar. "This is fundamental for appreciating the relative contributions of the different perturbations in relation to one another.

The Chromomap package we used for this analysis does not allow setting of scale bars. So instead we constructed heatmaps arranged by chromosomal coordinates and using the same scale. Both analyses are now shown in the revised version of Supp. Fig 13.

Minor comments:
- For figures 2, 3, 4, the legend should indicate whether a single focal plane (of the many stacks they acquired) or a projection is shown. If single plane, how was it selected? In the middle? How do they guarantee picking unbiasedly? If projection, was it sum of stacks or maximum intensity or..?

All images are maximum intensity projections of whole nuclei taken with 0.2 micron Z-spacing. This information is also in the “Microscopy” section of the Methods section. The information is now added to the figure legends. As explained in the Methods section, quantification was performed on the image stacks, rather than on the maximum intensity projections.

- Fig1B: the titles of the graph have different size

Fixed.
- Line 273: a space is missing between In and TIR1

Fixed
- Figure 3C and D: the asterisks are misaligned

Fixed.
- For fig 3D the description on the Y axis signal/background is unclear and lacking critical information. Is it mean fluorescence intensity? Maximum fluorescence intensity? Mean should be used.

The graph shows mean fluorescence intensity in the nucleus/background. The label has been changed. Same change in labels was made on Figs 2 and 4 for similar graphs. The clarification was also added to the figure legends by adding the word “mean”.

- Line 389, please add “at least at the resolution scale of our Immuno Fluorescence assay”. Without other assays (e.g., ChIP) the authors cannot rule our effects of CEC-4 on H4K20me1.

Added.

- line 402, unclear what they mean with more diffuse with respect to the X in cec-4 mutants: less compact? If so, state that.

The word was changed to “less compact”

- Line 454, the term movement is not accurate as the authors do not perform any time lapse imaging. Please, revise.

Revised to “did not significantly change X localization”

- Line 784 “cec-4” should be italicized

Done.

Reviewer #2: Trombley et al. have made substantial revisions to their original manuscript to improve the robustness, transparency, and quantitative nature of their conclusions. My concerns have been largely satisfied (except one point below), and I find the choice of revisions by the authors reasonable.

My remaining concern is the interpretation of the CEC-4 results, which the authors can clarify with changes to the text. Since their previous results showed that the left arm of the X (where CEC-4 binds H3K9me) is not detached from the lamina in cec-4 mutants, and the current manuscript does not present results to contradict this, I believe the conclusions should be modified. As the other reviewer pointed out, loss of cec-4 could result in the decompaction of chromatin without loss of tethering. Moreover, the authors did not provide evidence that the H3K9me binding activity of cec-4 is required for the observed change in chromosome territory morphology. Some uncharacterized function of cec-4 independent of H3K9me binding on the left arm, or downstream of cec-4, may be aiding the compaction of the X globally. I do not think additional experiments to investigate this aspect are necessarily within the scope of this study. Therefore, the authors should avoid statements that link the phenotypes observed to K9me binding or lamina tethering and instead use more accurate descriptors like 'overall localization', 'territory volume/compaction', or similar.

As detailed above, these changes to the text were made.

Instances where corrections to cec-4 interpretation are needed:
- title 'perinuclear tethering'
- line 46
- line 170
- line 649

All these sentences were modified.

Additional point: Include the degree of X gene derepression in the abstract (line 42) or state 'partial derepression' to avoid misleading readers into thinking the Xs are completely derepressed.

We added the degree of derepression in the abstract.
