# Supplementary material for: Condensin IDC, DPY-21, and CEC-4 maintain X chromosome repression in C. elegans
Source: PLoS Genet. 2025 Apr 9;21(4):e1011247. doi: 10.1371/journal.pgen.1011247 (PMC12013946; doi:10.1371/journal.pgen.1011247)
Supplement: Attachment — Submitted filename: Response 2.docx [file pgen.1011247.s031.docx]

Dear Editors,

The previous version of our manuscript was returned to us with the decision “minor revision”. The of decision letter stated:

“As you will see, there are multiple reviewer concerns listed, although all these can be addressed by changes to your text (i.e. no new experiments are required). Specifically, the data do not support the claim of perinuclear tethering and therefore we ask that you change or soften your claims that localization or tethering to the perinuclear region is related to compaction. Please also see other specific points in the reviewer comments. I do not expect a revision to have to go out for review again if you are able to make these changes to your text.”

There were no reviews attached, and upon asking for clarification, an editor told us that we just need to tweak the language in the manuscript text. However, we were somewhat unsure about what specific changes we needed to make and in which sentences.

In the previous version of the manuscript, we already changed the title and the text throughout to only make conclusions about CEC-4 activity, and to avoid making any conclusions about perinuclear tethering. In fact, the word “perinuclear” is not found in the manuscript, except in the reference list in the title of a paper about CEC-4. The word “tether” appears a few times in the introduction with reference to CEC-4’s known role in tethering heterochromatin to the nuclear lamina. The word also appears in the discussion, where (according to the previous decision letter), we were allowed to “suggest [this role] in a hypothetical model”.

For the current revised manuscript, we made a few additional tweaks to the text, to rephrase our observations, when possible, without using the word “tether”. The word now only appears in the manuscript 4 times, three times referring to ref 32 (a paper describing CEC-4’s role in perinuclear anchoring), and one additional time in the discussion when suggest that the changes we see in X morphology may “potentially” be related to CEC-4’s role in tethering H3K9me3 chromatin to the nuclear periphery.

Regarding the claim that localization or tethering to the perinuclear region is related to compaction:

This suggestion in the previous revision was added in response to a reviewer’s comment. The reviewer pointed out that “it is possible that the X chromosome, visualized with a whole chromosome paint, remains bound to the nuclear lamina but due to decompaction, reaches at least partially the internal zone of the nucleus.” To acknowledge this, we inserted the following sentence into the manuscript: “We note that some of the apparent relocation toward center could be a secondary consequence of decondensation” (Lines 461-462). To which the reviewer replied “I appreciate the authors discussing the role of decondensation.” To further clarify what was meant by our statement, we now changed it to “We note that some of the apparent relocation toward center could be a secondary consequence of decondensation, which allows the chromosome to occupy more internal regions of the nucleus.”

If additional changes need to be made to the text, it would be very helpful to know which specific sentence(s) needs to be changed.

(We also made a change in response to a journal requirement. We referred to panels Fig S13 incorrectly. After we made changes to the figure, we did not change the references to the figure in the text appropriately. This error is now fixed.)
